# Digital Monitoring and Modeling of Honey Bee Foraging on Clover Flowers Under Heat Stress

**DOI:** 10.17912/micropub.biology.002378

**Published:** 2026-08-27

**Authors:** Peter Oliver, Sushma Naithani, Joussy Hidrobo-Chavez, Yashoda Murali, Benjamin Nichols, Ramesh Sagili, Pankaj Jaiswal, Yue Zhang

**Affiliations:** 1 School of Electrical Engineering and Computer Science, Oregon State University, Corvallis, OR, United States; 2 Department of Botany and Plant Pathology, Oregon State University, Corvallis, OR, United States; 3 Department of Horticulture, Oregon State University, Corvallis, OR, United States

## Abstract

Plant-pollinator networks are increasingly threatened by rising global temperatures, yet monitoring these interactions at scale remains challenging. This study presents a digital monitoring framework that leverages artificial intelligence (AI) and machine learning (ML) to analyze honey bee ( *Apis mellifera* ) foraging on white clover ( *Trifolium repens* ) under normal and heat-stress conditions. The main objective was to establish a proof-of-concept for real-time, AI-driven pollinator monitoring rather than to draw definitive conclusions about the effects of heat stress on honey bee foraging behavior. This advancement supports smart farming decisions, such as optimizing clover buffer placement and adaptive honey bee management under heat stress.

**Figure 1. Impact of heat stress on white clover ( Trifolium repens ) pollens and on honey bees ( Apis mellifera ) foraging:** (A) Dissection of a white clover floret. (B) Impact of heat stress on clover plants in an open field. (C) Microscopic images of freshly collected and stained pollen (upper panel) and pollen in the germination solution for 24 hours (bottom panel) are shown from plants grown at normal day temperature <30°C and under hot day temperature >35°C for 3 days and 4 days. (D) Experimental setup in a greenhouse to digitally monitor honey bees' foraging activity. Number of honey bee visits to labeled clover flowers for selected dates across three consecutive weeks of varying temperature: (E) week 1, max temperature >30°C; (F) week 2, max temperature <30°C; (G) week 3, max temperature >30°C.

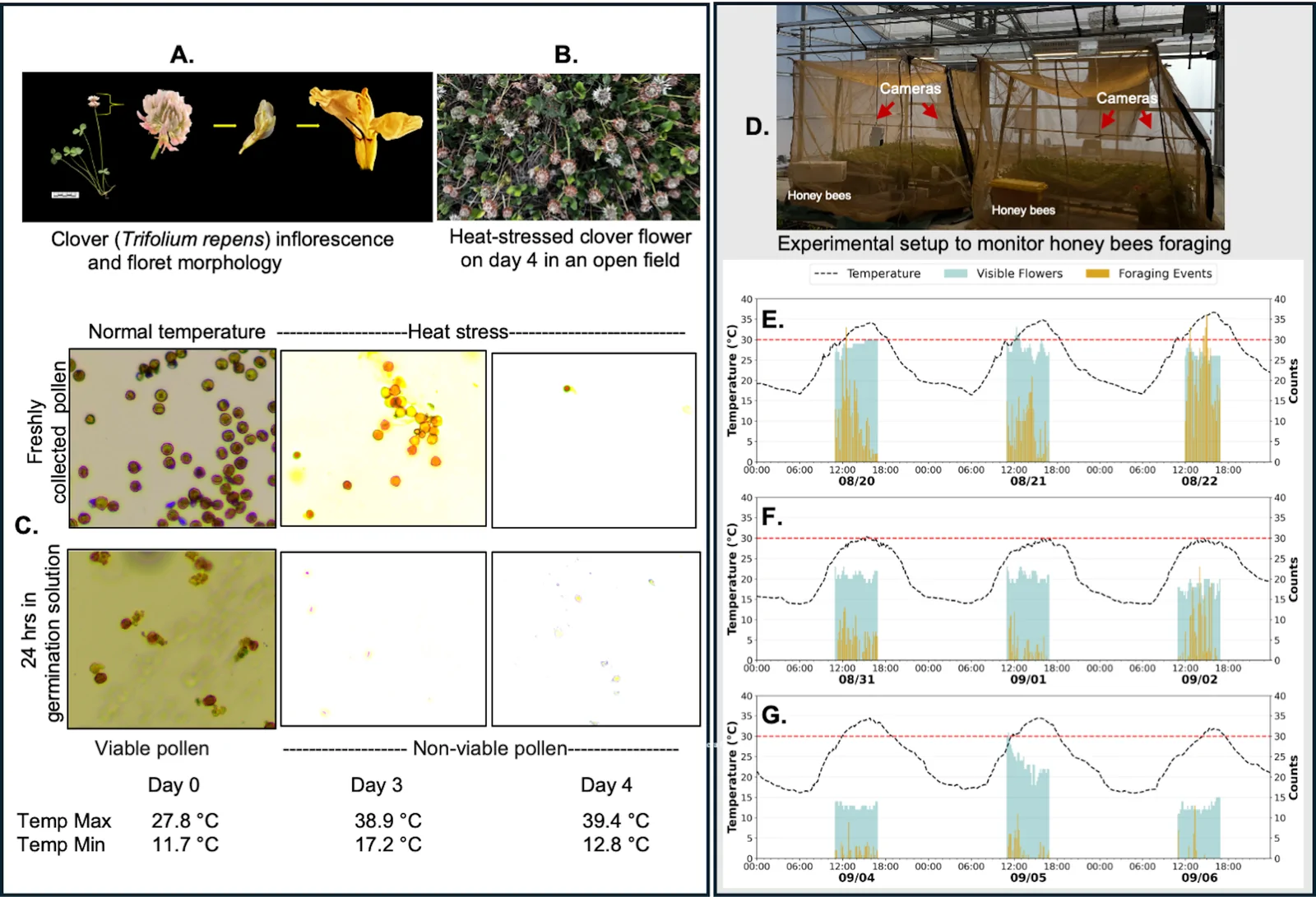



## Description

Pollination relies on complex, dynamic interactions between plants, pollinators, and their environment. Rising global temperatures are increasingly disrupting plant–pollinator mutualisms (Hegland et al. 2009; Layek et al. 2025; Marten-Rodriguez et al. 2025; Walters et al. 2025). Heat stress triggers physiological disruptions in plants, leading to reproductive failures and poor nectar quality (Defalque et al. 2025), while simultaneously diminishing the olfactory signals required for pollinator attraction (Cordeiro and Dotterl 2023a; Cordeiro and Dotterl 2023b; Nooten et al. 2024). Pollinators' fitness is affected directly by thermal stress and indirectly by stressed host plants (Walters et al. 2024). Furthermore, rising temperatures advance flowering before pollinator emergence, heightening the risk of secondary extinction due to nutrient limitation (Balasubramanian et al. 2006; Peng et al. 2025). Despite growing recognition of the decline of pollinators in response to climate change (Defalque et al. 2025; Lohani et al. 2020; Mehmood et al. 2025; Walters et al. 2024) and its impacts on ecosystem functioning and agriculture, scalable tools for real-time monitoring of plant-pollinator interactions are lacking.

Digital monitoring using artificial intelligence (AI) and machine learning (ML) is gaining attention across agricultural applications (Kamilaris and Prenafeta-Boldú 2018), including bee counting and tracking (Bjerge et al. 2022; Chiranjeevi et al. 2025; Gernat et al. 2023; Odemer 2022; Ratnayake et al. 2024; Ratnayake et al. 2023; Ratnayake et al. 2021; Stark et al. 2023). Initially, AI/ML-based studies of honey bees were conducted in laboratory settings with fixed backgrounds, lighting, and bee orientation. Gernat et al. 2023 used a custom convolutional neural network (CNN) to track trophallaxis between barcoded bees in a hive, using a high-resolution infrared machine vision camera. Rozenbaum et al. 2024 used a You Only Look Once (YOLO) CNN to track honey bee foragers' movements in a planar maze, with a camera at a high image-capture frame rate. In contrast, monitoring honey bees' foraging in uncontrolled environments is challenging due to variable backgrounds, shifting illumination/vegetation, and the impracticality of high-performance cameras for in situ studies. A few studies have successfully employed off-the-shelf cameras for in-situ insect detection. Bjerge et al. 2022 proposed a classification and tracking algorithm using a YOLOv3 model, achieving an average precision of 89% for tracking eight insect species. Ratnayake et al. 2021 remotely recorded 30 fps video footage of freely foraging, unmarked insects and used a YOLOv4 hybrid model to detect strawberry flowers and four insect classes, including honey bees, accurately detecting 97% of honey bee-flower interactions.

 Here, we report AI-ML-supported digital monitoring of honey bees foraging on white clover under normal and heat-stress conditions (see Figure 1).

White clover, with basal thermotolerance (Liu et al. 2021), maintains more stable nectar secretion and pollen viability than heat-sensitive floral resources under heat stress (Norris 1987; Walters et al. 2024). Previously, white clover flowering was reported to peak under long-day conditions (16h day/8h night) at 30°C (Norris 1987). We compared pollen quality of plants grown under ambient daytime temperatures (<30°C) with those plants grown under high temperatures (>35°C) using standard methods (Benner and Townsend 1973; Stokes and Geitmann 2025). As shown in Figure 1C, after three days of heat exposure, the white clover pollen count decreased to 80%, and all pollen became non-viable. After four days of heat stress, the anthers lost their structural integrity, the pollen count declined to <1%, and viability declined to 0% ( Figure 1C ). However, one day of heat exposure (max 38.9°C, min 13.9°C) did not significantly affect pollen counts or viability, while two days of heat exposure (max 38.3°C, min 15.0°C) reduced viable pollen count by 50%. The severity of heat stress increased with prolonged exposure to ≥35°C, culminating in complete floral failure within 72 hours. These findings are consistent with previous reports showing white clover flowering decline sharply above 30°C (Norris 1987), that pollen is among the most thermally sensitive tissues in flowering plants (Jiang et al. 2019), and that 32°C to 35°C marks a common threshold for pollen viability loss across plant taxa (Chen et al. 2020; Fragkostefanakis et al. 2016; Hu et al. 2021; Jegadeesan et al. 2018; Kumar et al. 2019; Pressman et al. 2002; Rosenberger et al. 2024; Zhang et al. 2021). Our results suggest that white clover serves as a reliable floral resource within a 30–35°C temperature range, when nutritional resources for bees become limited.

Honey bees, the principal pollinators of white clover, are vulnerable to thermal disruption. Typically, within honey bee colonies, brood temperature is maintained between 33°C and 36°C. Deviations from this thermal optimum cause developmental deformities and increase bee mortality (Bordier et al. 2017; Groh et al. 2004; Tautz et al. 2003). As ambient temperatures rise, honey bees shift from nectar/pollen foraging to water foraging and nest-cooling (Bordier et al. 2017). Thus, temperatures >35°C represent a critical "tipping point" at which plant reproductive success, pollinator efficiency, and health collapse simultaneously. We chose a temperature range of 30°C to 35°C to assess heat's impact on honey bees' foraging, using two net enclosures in a semi-controlled greenhouse. Each enclosure contained three trays of clover plants (~300 plants total), a honey bee nucleus colony, and two cameras ( Figure 1D ). Greenhouse conditions were set to 25°C day/15°C night for the normal temperature, and 35°C day/15°C night for the heat treatment. Mostly, honey bees were active after temperatures exceeded 25°C, and maximum foraging occurred between 12:00 and 16:00 hours. Our cameras captured images at 5-second intervals consistently between 12:00 and 17:00 hours over 21 days. A total of >280,000 images were captured.

We chose the YOLO architecture (Stark et al., 2023) to detect and classify honey bee- flowers interactions from captured images. Our cameras captured a much wider field of view, making individual bees difficult to detect because of their small size. Thus, we used a two-stage method: a YOLOv11 detection model to locate identifiable flowers, followed by a YOLOv11 classification model to check each flower for honey bees. Using test subsets of the captured images, our model achieved 95% precision in detecting flowers and 98% accuracy in classifying whether a honey bee was present on a given flower (a true foraging event). Given a positive classification in a captured image, we checked the two previous images (taken 5 and 10 seconds prior) for flowers detected in the same location (bounding boxes with <20 pixels of difference in all coordinate dimensions), and counted it as a foraging event only if the previous classifications were negative. We did this to avoid double-counting of foraging events by a single honey bee that lasted longer than 10 seconds. A limitation of this approach is its inability to detect unique consecutive foraging events lasting less than 10 seconds by different bees on the same as well as unique foraging events on flowers located very close together. For data visualization ( Figure 1E- 1G), we aggregated the numbers of detected flowers and foraging events (from both enclosures) into 10-minute intervals to match the recorded temperature data from the HOBO temperature data logger. The flower counts were taken as the mode of detected flowers in the images during the interval, and the foraging counts were taken as the sum of all foraging events observed during the interval.

During the first week, we observed the highest number of flowers (24 and 33) and the highest foraging. Honey bees foraged optimally between 12:00 and 16:00 hours, and their activities declined when temperature increased >30°C ( Figure 1E- G). In the second week, foraging was recorded for flowers ranging between 15 and 23 ( Figure 1F ). The greenhouse temperature remained <30°C, and honey bees foraged throughout the day, suggesting that heat, rather than colony behavior, is the cause of the decline in foraging. After four days, we repeated the heat-exposure experiment with daily high temperatures between 32.0 °C and 34.5°C and flowers ranging from 11 to 18. As shown in Figure 1G, foraging was relatively low at temperatures >30°C. We observed significant honey bee mortality. The three-week honey bee population decline aligns with worker bees' lifespans: workers transition to foragers around day 21 and spend one to three weeks collecting nectar and pollen (Robinson 1992). In addition, the constraints of the enclosed experimental setup and heat stress directly contributed to high honey bee mortality. However, we also find a plausible, though indirect, link between diminished pollen in plants, fewer flowers, and reduced honey bee foraging. This is consistent with the fact that clover pollen is a key nutritional resource for honey bees, and foragers assess pollen quality through direct contact (texture, moisture) and chemical cues; heat-stressed flowers likely become progressively less rewarding with repeated visits.

Our results provide a proof-of-concept for the real-time monitoring of plant-pollinator interactions. Such advances will inform smart farming decisions, helping farmers strategically deploy floral resource provisioning and proactively manage honey bees. Although this study is based on honey bees and a relatively uniform stand of white clover, the general approach could be applied to more diverse flower and pollinator communities. However, greater variation in flower morphology, vegetation structure, density, occlusion, and background may reduce the performance of detection and classification models. Extending our framework to diverse communities would require additional training data representing relevant species.

## Methods

**Pollen counts and viability assay**

We compared flowers of white clover grown in an open field under ambient daytime temperatures (ranging 25–30°C) with those exposed to high daytime temperatures (ranging 35–42°C) by directly assessing pollen germination capacity as a proxy for viability using standard methods (Benner and Townsend 1973; Stokes and Geitmann 2025). Three globular inflorescences of white clover (consisting of 25-30 florets) from each set were examined for the presence of viable pollen for five days (n = 30 inflorescences total). Pollen grains were collected from ten fully open florets (from each inflorescence) by removing the anthers and immersing them in germination solution, with gentle agitation to release the pollen grains. Twenty microliters of the pollen suspension were transferred to a microscope slide and immediately stained with Lugol solution (Sigma-Aldrich, St. Louis, MO, USA) to view fresh pollen; the remaining suspension was incubated at 25°C for 24 hours, then stained with Lugol solution to assess germination capacity. All slides were examined under bright-field illumination at 25x magnification using an ECHO Rebel microscope (Discover Echo Inc., San Diego, CA, USA), with pollen counts and germination tube formation assessed across a minimum of three fields of view per slide. Fresh pollen from normal flowers stained dark blue, while heat-stressed pollen grains stained yellow or brown. For the viability assessment, grains producing a visible pollen tube after 24 hours in germination solution were scored as viable, while grains lacking a germination tube were scored as non-viable.

**Honey bee colony establishment and maintenance**

Two small portable honey bee ( *Apis mellifera* ) colonies housed in cardboard boxes (referred to as nucleus colonies) were established using standard protocol (Sagili et al. 2015). Each nucleus colony consisted of one frame of emerging bees, two frames with approximately a 1:1 ratio of unsealed and sealed brood, one frame of honey with adhering bees, and one empty frame with drawn comb from donor colonies. Each nucleus colony had a laying queen and approximately 6,000 adult bees. Adult bees were shaken into the nucleus colonies from open brood frames of the donor colonies. These nucleus colonies were established at the Oregon State University apiary at the Oak Creek Center for Urban Horticulture, then brought to the greenhouse and placed inside the experimental net enclosures.

**Plant growth, maintenance, and experimental setup**

White clover ( *Trifolium repens* ) seeds were surface-sterilized, soaked in water, and vernalized at 4°C for 3 days prior to sowing to promote uniform germination. Seeds were sown into a commercial Sunshine soil mix (Sun Gro Horticulture, Agawam, MA, USA) in 12-inch pots. Prior to sowing, the soil was watered to saturation to ensure adequate moisture for germination. Pots were placed in large trays and sub-irrigated throughout the growing period, allowing water to flow from the trays into the soil without disturbing the seedlings or displacing the seeds. Plants were maintained under a 12-hour light/12-hour dark photoperiod, with daily temperatures set at 25°C during the day and 15°C at night. Maximum germination was observed within four days. The five-week-old seedlings were transplanted into three large trays, each containing 100 plants. These seedlings were maintained under a 16-hour light/8-hour dark photoperiod, with daily temperatures set at 25°C during the day and 15°C at night for another three weeks to stimulate flowering. Plants were watered every third day between 8:00 and 9:00 hours throughout the experiment.

To investigate the impact of high temperatures on honey bee foraging on clover flowers, we constructed two net enclosures ( Figure 1D ), each housing two cameras mounted on an internal wooden bar to capture a full view of the clover plants. Finally, eight-week-old flowering clover plants and a honey bee nucleus colony were placed in the net enclosure to facilitate controlled pollinator-plant interactions. Given the limited number of clover flowers available relative to colony foraging demand, 200 ml of a light sugar syrup (40% w/v sucrose) was provided constantly via a hive-top feeder to support honey bee health and maintain colony activity throughout the observation period. A 40% w/v sucrose syrup was selected because it is commonly used to sustain bees in cage studies and is more diluted than the standard sucrose solution used in standard colonies. This sucrose concentration in the syrup did not discourage bees from foraging on available flowers, thereby minimizing potential bias in foraging behavior toward the clover plants under study (Chakrabarti et al. 2020; Moreno and Arenas 2023). Honey bee foraging on clover flowers was assessed under both normal temperature (25°C day/15°C night) and heat-stress conditions (35°C day/15°C night). A HOBO MX1101 data logger (Onset Computer Corporation, Bourne, MA, USA) was placed adjacent to the experimental setup to continuously record temperature at 10-minute intervals throughout the experiment.

**Digital monitoring of honey bee foraging**

Two GoPro HERO8 Black (GoPro, Inc., San Mateo, California, USA) action cameras (C1 and C2) with 12-megapixel sensors were installed inside the net enclosure approximately 45 cm above the tops of the clover plants, as shown in Figure 1D . The two cameras were aimed directly downward and positioned so that each captured half of the clover bed. The cameras were set to use the wide-angle lens with an output image resolution of 3000x4000 pixels. The cameras captured images at 5-second intervals consistently between 12:00 and 17:00 hours over 21 days in August and September of 2025 while the honey bees were actively foraging.

**Data analysis and visualization**

Of the >280,000 captured images, 177 images were labeled with bounding boxes around each visible clover flower for a total of 2097 labeled clover flowers. To capture variation across the observation period, images for labeling were selected by taking a minimum of three images from each camera on each day, with at least one hour between images. The bounding boxes ranged in size from 30 to 100 pixels in each dimension. These labels were used to perform transfer learning on the pretrained YOLO11 object detection model, which has 25.3M parameters and is available through the ultralytics Python package ( https://github.com/ultralytics/ultralytics ). Due to the small size of the clover flowers in the images, they were scaled to a relatively large maximum dimension of 2048 pixels during training. The 177 labeled images were randomly divided into training and test sets using a 75/25% split. Model performance was evaluated on the held-out test set of 44 images, in which a predicted bounding box was considered a true positive if its intersection-over-union (IoU) with a ground-truth bounding box exceeded 0.5. The model achieved its best performance after 41 epochs with a precision of 0.922, recall of 0.919, F1-score of 0.921, mean average precision of 0.953 at an IoU threshold of 0.5 (mAP50) and 0.805 when averaged across IoU thresholds from 0.5 to 0.95 (mAP95). Because the training and test images originated from the same image collection, the test set evaluates generalization to previously unseen images from the same experimental setting rather than to an entirely independent image collection. In particular, images from the same camera and observation days may share environmental and scene-specific characteristics, which could result in some correlation between the training and test sets despite the temporal spacing of the selected images. Therefore, the test performance should be interpreted as within-dataset generalization and may not fully represent performance on images collected under different environmental conditions or camera configurations, such as with different types of plants and flowers.

After training, we used the model to detect clover flowers in every collected image, creating image cutouts around each flower that was detected with >75% confidence. We added 20 pixels of padding on all sides to the image cutouts to account for possible honey bees on or around the flowers. These image cutouts were then passed to the InsectNet, a pretrained insect classification available on GitHub ( https://github.com/ShivaniChiranjeevi/Insect-Classifier ). This model has previously supported a mean-per-class accuracy (MPCA) of 96% for classifying 54 prevalent insect species in the US Midwest region (Chiranjeevi et al. 2025). For our data, we accepted classifications for any species in the Apidae family as positive detections of honey bees. We then manually verified the classifications for ~5,700 clover flower cutouts, labeling them as either containing a honey bee (foraging event) or not containing one (no foraging). We used these labels to train a second YOLO11 classification model with 12.9M parameters, which is also available as a pretrained model through the ultralytics package. We randomly divided the labeled cutouts into training and test sets following an 80/20% split. Using a batch size of 32, the classification model achieved its best performance on the held-out test set after 34 epochs, with a precision of 0.986, recall of 0.993, F1-score of 0.989, and accuracy of 0.989. The training and test images were taken from the same collection of labeled cutouts, which could again result in correlation between the training and test sets, so the test performance may not generalize to image cutouts involving different flowers, pollinators, or experimental conditions. Training and evaluation using independent image collections would provide a stronger assessment of the model's broader generalizability.

Finally, we applied our clover flower detection and foraging event classification models in a two-stage pipeline to obtain counts of clover flowers and honey bee foraging events for each original image. In the first stage, the YOLO11 detection model is used to count the number of visible clover flowers, and in the second stage, the YOLO11 classification model is used to decide whether a honey bee is present on each of the detected clover flowers. The Python scripts used for data analysis are available at the Naithani Lab GitHub: https://github.com/naithanis/Naithani-lab-codes/tree/master/Clover-honey bees .

## Reagents

White Clover ( *Trifolium repens L.* )

Honey bee ( *Apis mellifera* )

Lugol solution: 0.33% iodine and 0.66% potassium iodide (L6146, Sigma-Aldrich, St. Louis, MO, USA).

Pollen germination solution: 25% sucrose (w/v), 100 ppm CaCO₃, and 50 ppm H₃BO₃ in deionized distilled water.
